# Simulation of Long-term Yield and Soil Water Consumption in Apple Orchards on the Loess Plateau, China, in Response to Fertilization

**DOI:** 10.1038/s41598-017-05914-9

**Published:** 2017-07-14

**Authors:** Xingxing Peng, Zheng Guo, Yujiao Zhang, Jun Li

**Affiliations:** 1 College of Agronomy, Northwest A & F University, Yangling, Shaanxi, 712100 China; 2 College of Forestry, Northwest A & F University, Yangling, Shaanxi, 712100 China

## Abstract

The Loess Plateau, China, is the world’s largest apple-producing region, and over 80% of the orchards are in rainfed (dryland) areas. Desiccation of the deep soil layer under dryland apple orchards is the main stressor of apple production in this region. Fertilization is a factor that causes soil desiccation in dryland apple orchards. Given its applicability and precision validations, the Environmental Policy Integrated Climate (EPIC) model was used to simulate the dynamics of fruit yield and deep soil desiccation in apple orchards under six fertilization treatments. During the 45 years of study, the annual fruit yield under the fertilization treatments initially increased and then decreased in a fluctuating manner, and the average fruit yields were 24.42, 27.27, 28.69, 29.63, 30.49 and 29.43 t/ha in these respective fertilization treatments. As fertilization increased, yield of the apple orchards increased first and then declined,desiccation of the soil layers occurred earlier and extended deeper, and the average annual water consumption, over-consumption and water use efficiency increased as fertilization increased. In terms of apple yields, sustainable soil water use, and economic benefits, the most appropriate fertilization rate for drylands in Luochuan is 360–480 kg/ha N and 180–240 kg/ha P.

## Introduction

Apples, which are widely planted on the Loess Plateau, are the main income-generating fruit tree, and apple orchards cover a planted area of more than 1.2 million ha across the Weibei Dryland Highlands of Shaanxi^1^. A total of 80% of these apple orchards are rainfed (dryland), and their intense water consumption due to apple transpiration has caused excessive soil water depletion and consequent deep soil desiccation. Furthermore, the excessive and widespread applications of nitrogen and phosphorus in apple orchards improve apple yield and quality but intensify deep soil desiccation and restrict the stable and healthy development of the apple industry in semi-humid regions^2–4^. Excess applications of nitrogen and phosphorus was observed in 74% and 54% of the apple orchards, respectively, in the Weibei Dryland Highland^5^. Management of N and P fertilization is pivotal for sustainable fruit production in North China^6^. Wang *et al*.^7^ found that in the semi-arid region of the Loess Plateau, excessive application of P could result in high P-fixation capacity and consequently, severely damaged environments. In terms of economic returns, Qinguan apples have a P-use efficiency that is 50% higher than that of corn^8^. Researchers in Zagora Pelion (Central Greece) found that in apple orchards, the N inputs were five times higher than the nitrogen outputs; thus, reductions in N fertilization were considered^9^.

Some researchers have carried out short-term field experiments to determine the effects of different fertilization treatments on fruit yield and the quality of apple. However, few long-term experiments have investigated apple yield, soil water use and soil desiccation under different fertilization treatments^4, 10, 11^. Several models have been proposed to monitor apple tree growth^12–14^, e.g., models for root water uptake and soil water recovery after apple tree harvesting. The Environmental Policy Integrated Climate (EPIC) model has been used as a simulation model of crop productivity systems for many crops and is a universal model for water and soil management as well as crop productivity evaluation^15–17^. Ko *et al*.^18^ found EPIC to be a useful method for managing irrigated cotton and maize. Michele^19^ used EPIC to simulate irrigation scheduling in sunflower and found that the model effectively compared management strategies. The soil water and yield dynamics of grain crop rotation systems in the semi-humid region of the Loess Plateau have been well described using the EPIC^20^ model. Previous model applications involving soil water and yield of apple orchards on the Loess Plateau of China indicate that the EPIC model accurately simulates apple production^21^. However, further studies are needed to simulate soil water and crop productivity in response to different fertilization regimes applied to apple orchards on the Loess Plateau.

Using the EPIC model, this study sought to quantitatively simulate yield fluctuations and soil desiccation under different fertilization treatments applied to apple orchards over a long period of time (45 years). The effects of fertilization on apple yield and deep soil desiccation were analyzed to determine the best fertilization regime relative to local precipitation and to promote sustainable soil water utilization. Hence, the main objective of this study was to guide fertilization management decisions for sustainable apple production in the Weibei Dryland Highland on the Loess Plateau and in similar regions of the world.

## Results

### Simulated yields in dryland apple orchards

The annual precipitation in Luochuan between 1965 and 2009 ranged from 343.50 mm to 899.60 mm and averaged 596.64 mm, with a standard error of 117.17 mm and a coefficient of variation of 19.64%. The precipitation decreased in a fluctuating manner and was 44.53 mm lower in the last 15 years compared to the first 15 years.

**Figure 1:**
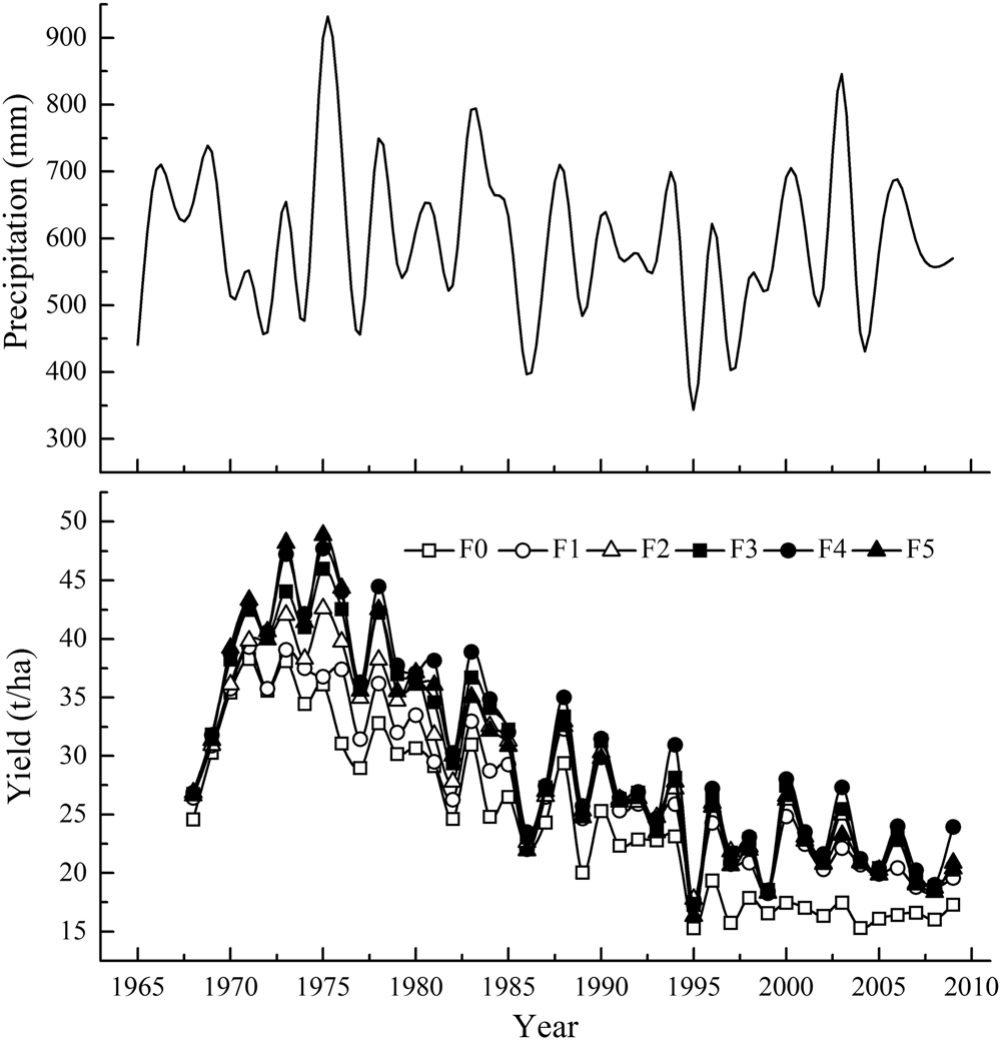
Simulated annual fruit yields under different fertilization treatments and precipitation in the apple orchards of Luochuan.

During the fruit-bearing stage of the apple trees (apple trees start to bear fruit at 4 years old, as simulated), the yields of 4- to 45-year-old rainfed apple orchards in the F0-F5 treatments tended to first increase to their maxima and then decrease in a fluctuating manner from 1968 to 2009 (Fig. 1). The yield curves of the different fertilization treatments generally peaked in the initial simulated years and in the rainy years and then exhibited a trough during the latter simulated years and in the dry years. The yields of orchards subjected to the respective F0-F5 treatments averaged 24.42, 27.27, 28.69, 29.63, 30.49 and 29.43 t/ha, with standard deviations of 6.14, 6.11, 6.83, 7.68, 8.12 and 8.36 t/ha. Compared to yields under F0, the yields under F1-F5 increased by 11.70%, 17.49%, 21.35%, 24.85% and 20.54%, respectively, which indicated that F3 and F4 performed the best. After 42 fruiting years, the yields decreased considerably under all fertilization treatments. The greatest decrease occurred under F0, and greater decreases occurred under higher compared with lower fertilization applications. The lowest yields occurred under the 6 fertilization treatments in 1995 when the precipitation was only 343.5 mm, and the highest yields under F0 and F1 occurred in 1971; under the other fertilization treatments, the highest yields occurred in 1975 when the precipitation reached 899.6 mm.

### Simulated drought stresses in rainfed apple orchards

**Figure 2:**
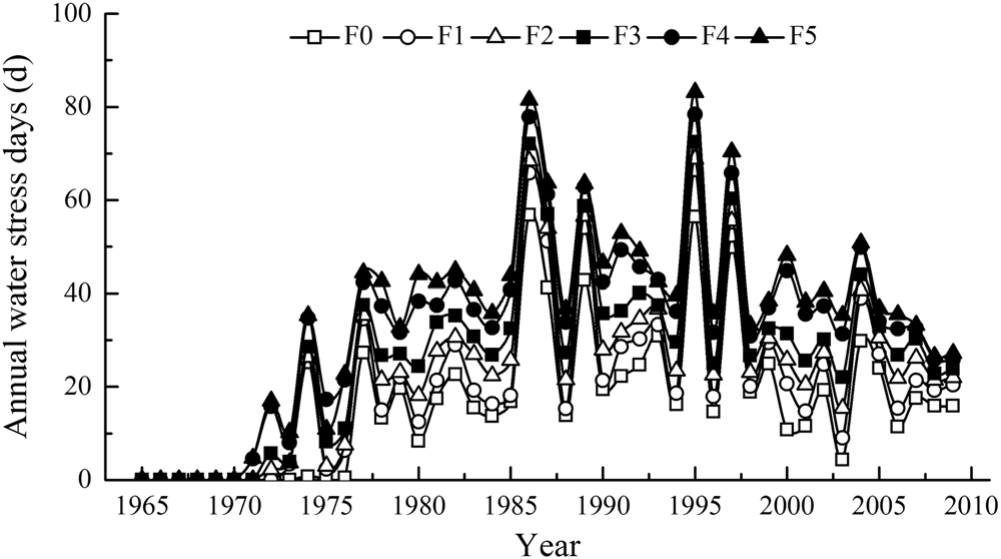
Simulated number of water stress days under different fertilization treatments in the apple orchards of Luochuan.

Drought stress is referred to as a restriction of crop growth due to water deficit, and in EPIC, its duration indicate its severity during the growing season. Over the 45 study years, the number of drought stress days under the respective F0-F5 treatments ranged from 0 to 57, 0 to 67, 0 to 69, 0 to 72, 0 to 78 and 0 to 83 days and averaged 16.71, 20.95, 24.01, 27.41, 33.06 and 35.12 days annually, with standard deviations of 14.84, 16.92, 17.41 18.24, 19.45 and 20.62 days. The number of drought stress days increased as the fertilization amount increased, and drought stress occurred earlier under higher fertilization. In the apple orchards, the earliest year of drought stress arising under the 6 respective fertilization treatments occurred in 1977 (in the 13-year-old orchards), 1974 (in the 10-year-old orchards), 1972 (in the 8-year-old orchards), 1972 (in the 8-year-old orchards), 1971 (in the 7-year-old orchards) and 1971 (in the 8-year-old orchards). The number of drought stress days under the different fertilization treatments tended to fluctuate in a basically identical manner, opposite from the manner in which the local precipitation varied. The peak number of drought stress days appeared in the dry years of 1986, 1995, 1997 and 2004. The number of drought stress days in the six different fertilization treatments peaked in 1995, reaching 56.39, 66.50, 68.83, 72.42, 78.40 and 83.14 days, in the F0, F1, F2, F3, F4 and F5, respectively, because precipitation in 1995 was only 343.5 mm (Fig. 2).

### Simulated monthly available soil moisture in the 0–15 m soil layer in the apple orchards

From 1965 to 2009, the simulated monthly available soil moisture in the 0–15 m soil layer in the respective F0-F5 treatments ranged from 723.22 to 2573.23, 699.88 to 2557.88, 681.28 to 2533.53, 669.60 to 2508.09, 663.72 to 2486.96 and 661.60 to 2479.27 mm and averaged 1267.40, 1223.44, 1191.33, 1153.71, 1116.45 and 1102.67 mm, with standard deviations of 556.9, 563.1, 555.2, 544.7, 525.3 and 521.1 mm and coefficients of variation of 43.9%, 46.0%, 46.6%, 47.2%, 47.0% and 47.2%. No obvious differences existed in available soil moisture between the high fertilization treatments (i.e., F4 and F5), but significant differences in available soil moisture occurred in the other treatments. As fertilization increased, the available soil moisture decreased and presented annual increases and seasonal fluctuations.

**Figure 3:**
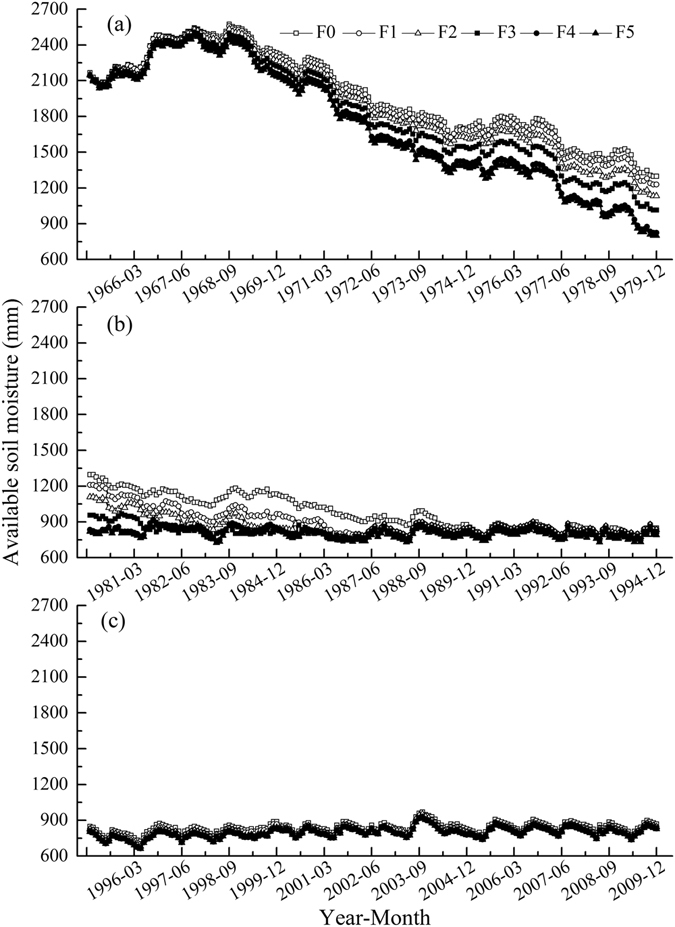
Simulated monthly available soil moisture in the 0–15 m soil level under different fertilization treatments in the apple orchards of Luochuan.

During the 45 simulated years, the monthly available soil moisture in the 0–15 m soil layer under the different fertilization treatments tended to vary similarly (Fig. 3). In the early stage of the 45-year simulation, the simulated monthly available soil moisture in the respective F0-F5 treatments tended to decrease clearly in a fluctuating manner before 1990, 1985, 1984, 1982, 1980 and 1980, with decreases at 1282, 1130, 1315, 1283, 1315 and 1345 mm and soil desiccation rates of 51.68, 58.57, 65.75, 75.47, 87.67 and 89.67 mm/a. Later, the monthly available soil moisture in the 0–15 m soil layer in the F0-F5 treatments varied at a lower level with seasonal precipitation. No obvious difference occurred in the soil desiccation rate between F4 and F5. In the Weibei Dryland Highlands, it frequently rains from July to September, resulting in a recovery of available soil moisture in most years.

### Simulated soil moisture distribution in the 0–15 m soil layer in apple orchards

**Figure 4:**
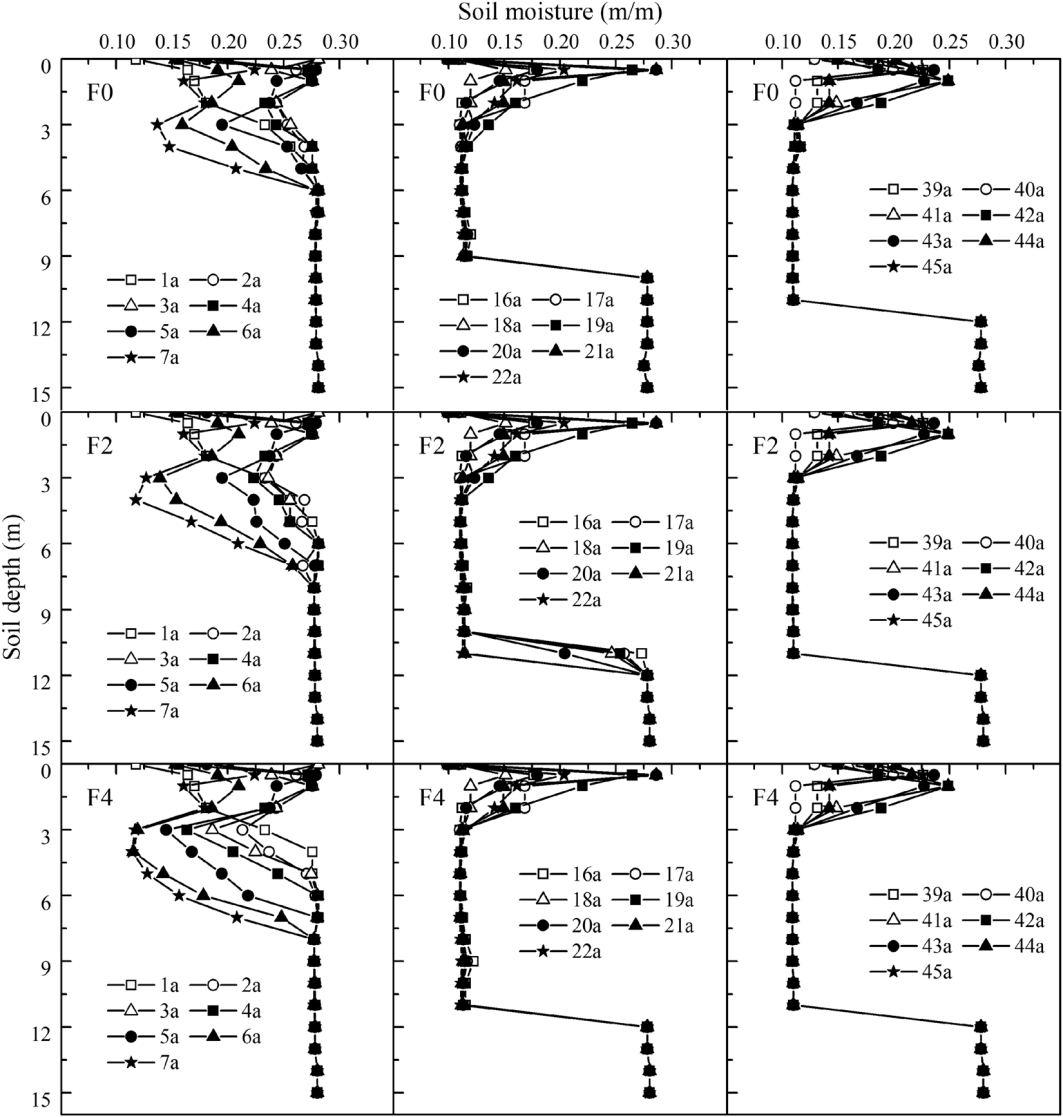
Simulated annual soil moisture profiles in the 0–15 m soil layer under different fertilization treatments in the apple orchards of Luochuan.

The soil moisture distributions in apple orchards during the initial (1965–1971), middle (1980–1986) and final (2003–2009) simulated years are shown in Fig. 4. The figure shows that there were approximately the same simulated annual soil moisture distributions in the 0–15 m soil layer under F0-F5. Compared with the soil moisture in the apple rhizospheres when the simulation began, in 1965, the soil moisture in the apple rhizospheres decreased yearly as the apple trees grew and as the dry soil layers appeared and then deepened and thickened each year until they stabilized. The dry soil layers in the respective F0-F5 treatments occurred in 1976, 1974, 1973, 1973, 1972 and 1972 and took 25, 23, 21, 18, 15 and 15 years to reach a depth of 11 m. As fertilization increased, the dry soil layers occurred earlier, accompanied by accelerated formation, and the depth of 11 m was reached earlier. Soon after the dry soil layers became stable, the effects of fertilization on the soil moisture in the apple orchards disappeared; the soil water at maximum soil depth was utilized by the apple trees; therefore, the field water consumption of the apple trees mainly relied on seasonal precipitation. There was considerable variation in soil moisture in the 0–2 m soil layer, which resulted from the influence of rainfall infiltration.

### Simulated water consumption in apple orchards

During the period from 1965 to 2009, the simulated average annual water consumption in the respective F0-F5 treatments in the dryland apple orchards in Luochuan ranged from 335.8 to 831.0, 334.8 to 846.1, 332.6 to 864.7, 332.7 to 876.3, 332.9 to 899.3 and 332.9 to 900.0 mm and averaged 623.2, 625.6, 627.8, 628.0, 628.5 and 629.7 mm, with standard deviations of 110.4, 115.3, 118.5, 120.0, 122.5 and 123.4 mm. In apple orchards with 45-year-old trees, the total increased water consumption in the respective F1-F5 treatments was approximately 108, 207, 216, 239 and 293 mm, i.e., annual increases of 2.4, 4.6, 4.8, 5.3 and 6.5 mm compared with the average water consumption under F0. The average annual water consumption in the orchards increased as fertilization increased.

**Figure 5:**
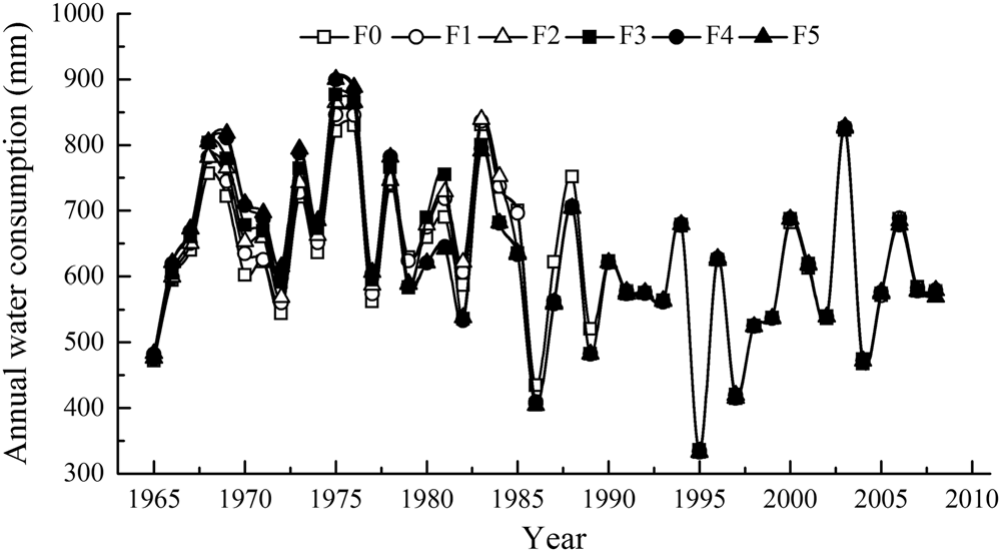
Simulated annual water consumption in the 0–15 m soil layer under different fertilization treatments in the apple orchards of Luochuan.

The water consumption differed significantly among the different fertilization treatments before the apple trees were 25 years old and was almost identical after the age of 26, although the values fluctuated with precipitation (Fig. 5). During the 45 years, the water use efficiencies in the F0-F5 treatments in the apple orchards of Luochuan were 39.19, 43.59, 45.70, 47.18, 48.51 and 46.74 t/(mm·hm^2^), respectively. These results indicate lower water use efficiency under F0 compared with the other treatments.

**Figure 6:**
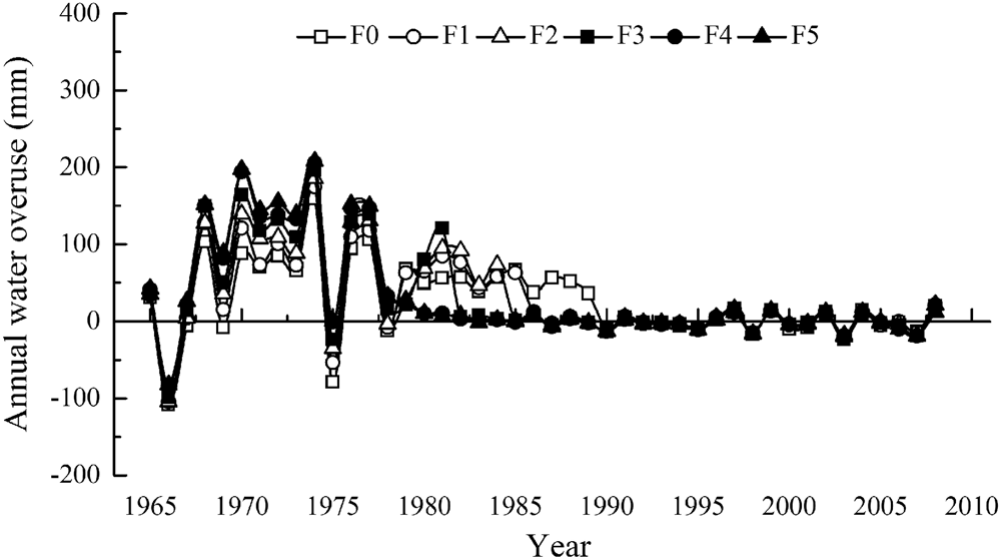
Simulated water over-consumption in the 0–15 m soil layer under different fertilization treatments in the apple orchards of Luochuan.

In the orchards, the annual soil water over-consumption was the difference between the annual water consumption and precipitation, a measurement of the soil water balance between soil water consumption intensity and soil water replenishment capacity from seasonal rainfall. The soil water over-consumption fluctuated around approximately 0 in the later stage of the 45-year simulation (Fig. 6). The soil water over-consumption in F1-F5 was 2.5, 4.6, 4.8, 5.3 and 6.5 mm higher, respectively, than that in F0. It follows from the standpoint of sustainable soil water utilization that F2 and F3 are the best choices, although under F4 and F5, the yields and water use efficiencies were higher, but soil desiccation occurred faster and more severely.

### Economic returns from apple orchards

Depending on the simulated yields and corresponding fertilizer rates under the different fertilization treatments, a regression equation was established between the yields and fertilizer rates as follows:

1 $${\rm{y}}=-2E-05{x}^{2}+0.0218x+24.692\quad {R}^{2}=0.98$$

In this equation, x is the application rate of N, and y is the yield of the apple orchard in question. The prices of nitrogen, phosphorus and apples were calculated depending on the prices and composition of the fertilizers. Because other management practices that accompanied fertilization were the same, their input costs were not taken into account, such that the overall economic returns of one apple orchard were calculated using the following equation:

2 $$Y=4y-4.5{X}_{N}-7.5{X}_{P}$$

In this equation, Y is the total economic return from the apple orchard, y is the yield of the apple orchard, X_N_ is the application rate of nitrogen, and X_P_ is the application rate of phosphorus. The overall economic returns from the apple orchard under the different fertilization treatments were calculated using this equation (Table 1). For comparison, we obtained the maximum economic returns and corresponding fertilization for the apple orchards. In Luochuan, the maximum economic returns were 117,985 yuan/hm^2^ orchard, and the corresponding orchard yield and nitrogen and phosphorus application rates were 30.49 t/hm^2^, 480 kg/hm^2^ and P 240 kg/hm^2^, respectively (Table 1).

**Table 1:** Economic returns from apple orchards under different fertilization treatments in Luochuan.

Fertilization	F0	F1	F2	F3	F4	F5
N kg/hm^2^	0	120	240	360	480	600
P kg/hm^2^	0	60	120	180	240	300
Yield	24.42	27.27	28.7	29.63	30.49	29.43
Economic Return	97672	108109	112776	11555	117985	112786

A suitable fertilization rate based on different statistical indicators of dryland apple orchards in Luochuan can be sourced from a comprehensive analysis of orchard yields, water use efficiency, soil water over-consumption and economic benefits (Table 2). In terms of orchard yield increases, sustainable soil water utilization and economic returns, the most suitable fertilization rates in dryland apple orchards in Luochuan were observed under F3 and F4, i.e., N 360–480 kg/ha and P 180–240 kg/ha.

**Table 2:** Recommended fertilizer amount for apple orchards in Luochuan based on different statistical indictors.

Indicators	Fertilization
Yield	F3-F5
Water use efficiency	F3-F4
Water over-consumption	F2-F3
Economic return	F4

## Discussion

### Dryland apple orchard yield and soil desiccation

The Weibei Dryland Highlands, a primary apple-growing region, has various meteorological conditions that are advantageous for apple growth and production. In this region, more than 80% of the orchards are rainfed—these orchards are not irrigated and are subjected to severe soil water over-consumption, marked by deep soil desiccation, soil impoverishment and low fertility. As a result, proper fertilization is very important for soil fertility maintenance, yield increases and sustainable soil water utilization^22^. Holb *et al*.^23^ reported that better mobility of artificial fertilizers gives rise to higher macronutrient uptake in apple orchards compared to organic orchards in Debrecen-Pallag, Hungary. Jun *et al*.^24^ found that nitrogen and phosphorus applied at rates of 250–300 kg/ha and 150–200 kg/ha, respectively, achieved an orchard target yield of 25–30 t/ha. In the current study, the best fertilization treatments were N 360–480 kg/ha and P 180–240 kg/ha, i.e., the nitrogen rate is at a level between its optimum and a practical level, and the phosphorus rate is equal to the optimum. This result likely occurred because in this study, the orchards were dryland orchards that were not irrigated and thus had low fertilizer utilization. Another reason for the large amounts of applied nitrogen was that no apple orchards were ever measured. Nitrogen can increase apple yields, but its excessive application will result in a series of adverse results, such as a deterioration in apple quality, extensive growth of tree components, decreased soil pH, soil nitrate accumulation and groundwater pollution^25, 26^.

Our results add to previous findings, i.e., that water and nutrients are the most important factors that influence the yields of dryland orchards, and fertilization can effectively improve water use efficiency and yields of dryland orchards but can easily increase water consumption through transpiration, resulting in increased yields accompanied by aggravated soil water consumption^27^. In our study, under high fertilization levels, the available soil moisture stabilized earlier and soil desiccation occurred faster; furthermore, no obvious difference in the soil desiccation rate was observed between F4 and F5. The available soil moisture markedly decreased in the middle stage of the 45-year simulation, and the dry years somewhat recovered and increased during years with high precipitation. In Luochuan, the soil water storage obviously decreased. The precipitation led to fluctuating decreases in the average annual water consumption because of the decreases and fluctuations in annual precipitation, in addition to the influence of increasing soil desiccation; and this was one of the main reasons for the fluctuating decrease in the apple yields. We found that fertilization increases can increase water use efficiency but may result in soil desiccation and soil water over-consumption in deep soil layers. Proper fertilization can improve fruit quality, reduce production costs, maintain soil fertility, eliminate nutrient deficiencies, and maintain tree vigor^28^. In addition to fertilization, the growth and yield of “Cortland” apples also depended on the weather conditions and tree age^29^.

This study demonstrated that the yields under F1-F5 increased by 11.70%, 17.49%, 21.35%, 24.85% and 20.54%, respectively, compared with the average yield under F0 in the 45 years of study. The yields increased obviously as fertilization increased, but F5, which had excessive fertilization, resulted in the wasting of resources and had an adverse influence on apple yield. Furthermore, soil desiccation occurred faster, and soil drought stress occurred earlier and was more severe with higher levels of fertilization. The thickness of the dry soil layers differed obviously among the different fertilization treatments before the apple trees reached 23 years of age. The dry soil exceeded a depth of 11 m in all treatments except the control, and soil moisture was close to the wilting point over a long-term period. In the apple orchards, the dry soil layers formed earlier and deepened faster with high fertilization compared to low fertilization. During the period of time when soil water decreased obviously, the soil desiccation rates in the fertilization treatments of F0, F1, F2, F3, F4 and F5 were 51.68, 58.57, 65.75, 75.47, 87.67 and 89.67 mm/a. Higher fertilization readily resulted in deep soil desiccation, which led to a fluctuation in soil water storage under lower level precipitation over a long-term period.

### EPIC simulation in this study

Recently, Li *et al*. developed a simulation verification and application of EPIC for the Loess Plateau, revealing dynamic water productivities of artificial forestlands and grain crops in dryland regions, providing new ideas for studying water productivity as well as the effects of soil water and fertilizer in apple orchards^30, 31^. Research on water productivity and soil water has mainly concentrated on short-term field experiments. Consequently, there is a lack of experiments that are continuously monitored over a long period of time. By combining a short-term field experiment with quantitative and dynamic simulations over a long-term period using WinEPIC, this study generated continuous dynamic data that could not be obtained using conventional research methods. In addition, the evolution of annual water productivities and deep soil water dynamics under different fertilization treatments in apple orchards was examined, thus providing a scientific basis for determining suitable fertilization treatments and growth years relative to local precipitation. This was a simple and effective quantitative research method. However, simulation differences occurred depending on the factors in different planting areas, the density of the apple trees, and the fertilization treatment. There were some differences between the simulated and practical growth conditions, which resulted from the omission of the effects of different site conditions, e.g., slope and aspect, in the simulation. In addition, we were unable to simulate the effects of orchard management measures on apple yields and soil water conservation, flower and fruit thinning, tree pruning and thinning, orchard grass planting and film mulching, etc. These defects might lead to differences between the simulated and measured results, although the simulation may still reflect water productivity and soil water variations under different fertilization treatments and may provide a scientific basis for soil water management and suitable fertilization. The simulation parameters must be further modified to more accurately simulate water productivity and the effects of deep soil desiccation.

## Conclusions

During the simulated period from 1965 to 2009, the apple yields under F0-F5 tended to first increase and then decrease in a fluctuating manner; as the fertilization level increased, drought stress occurred earlier and was more intensive. The monthly available soil moisture in all of the fertilization treatments presented an obvious fluctuating decrease and then varied at a lower level with seasonal precipitation. As fertilization increased, drying of the soil layers occurred earlier and deepened more rapidly; and the the soil dry layers reached their maximum depths earlier, and the average annual water consumption, soil water over-consumption and water use efficiencies increased. In terms of yield increases, sustainable soil water utilization and orchard economic returns, F3 and F4 were the best choices. Therefore, a suitable fertilization rate for dryland apple orchards in Luochuan is N 360–480 kg/ha and P 180–240 kg/ha.

## Materials and Methods

### Study location

The simulation study was conducted in apple orchards in Luochuan, a county in Shaanxi, which is located in the central area of the apple production region on the Loess Plateau and in the northeastern part of the Weibei Dryland Highland (109°13′14″—109°45′47″E, 35°26′29″—36°04′12″N). With a warm temperate and semi-humid continental monsoon climate, the county has an average altitude of 1072 m, an average annual precipitation of 600 mm, an average annual temperature of 9.2 °C, and an average frost-free period of 167 d; the rainy season coincides with high temperatures. Luochuan has a broken topography typical of the high plain and gully loess regions of the Loess Plateau, which is characterized by uniformly textured Heilou clay soil and very thick loessial soil that can exceed a depth of 100 m. In Luochuan, most apple orchards are rainfed, i.e., they are not irrigated.

### WinEPIC Profile

The Environmental Policy Integrated Climate (EPIC) model includes weather simulation, hydrology, eroded sediments, nutrient cycling, pesticide rate, crop growth, soil temperature, soil tillage, economic benefit and crop environmental control. Based on integrated growth parameters of more than 120 types of field crops, pastures, and forests, as well as apple trees, the EPIC model can be employed to simulate long-term dynamic changes in soil water and nutrient utilization as well as crop productivity on a daily basis. Furthermore, EPIC has been used to evaluate agro-ecological system management and its effect on soil and water resources^32, 33^. WinEPIC v. 3060, which was adopted in this study, is a new EPIC model that can be run on Windows with a user-friendly interface. Using WinEPIC, soil water dynamics can be described in more detail; the daily soil moisture that is thus obtained for different soil layers can be used to study crop production systems and, in particular, can be used to simulate ecological environmental effects related to soil moisture in drylands^34^. Our research team has simulated the biomass and soil desiccation effects of artificial *Robinia pseudoacacia* and Chinese pine forestlands on the Loess Plateau and has preliminarily investigated the yield and soil water dynamics in apple orchards on the Weibei Dryland Highlands^35–37^. The details of mathematical methods used for crop productivity as well as soil water and fertilizer movement have been reviewed in related literature^38–42^.

### Dataset construction for WinEPIC

WinEPIC was run with datasets comprised of local daily weather variables, soil physical and chemical properties, crop growth parameters and the management measures of apple orchards. Adapted from real-time weather data observations (1965~2009) from the Luochuan weather station, the daily weather variables mainly included daily solar radiation (MJ/m^2^), maximum temperature (°C), minimum temperature (°C), precipitation (mm), relative humidity (%), and wind speed (m/s). These daily weather data were input into WinEPIC in the EPIC format; then, a daily weather database for Luochuan was constructed^43^. The soil physical and chemical properties, which consisted of more than 40 terms relating to 0–15 m deep Heilu clay soil in Luochuan (Table 3), were adapted from soil census data such as those contained in the Records of the Chinese Soil Survey, the Soil of Shaanxi^44, 45^. These data were transformed into a typical soil parameter database of the research region in EPIC. On the Loess Plateau, soil water more than 10 m deep is typically used by rainfed orchards, and in some rainfed orchards, water that is nearly 15 m deep can be used. The maximum soil depth in this study was set to 15 m to examine the deep soil water utilization of the apple trees in the orchards. There was a relatively uniform soil texture at a depth of 0 to 15 m, unlike the other soil layers. To facilitate the analysis of soil moisture dynamics, the soil profile in question was divided into 17 soil layers: 0–0.01 m, 0.01–0.5, 0.5–1 m and 1-m-thick layers that were distributed between 1 m and 15 m deep. Some important physiological and ecological parameters of apples were modified in EPIC (Table 4) in reference to related research and observed data from the Loess Plateau^46^.

**Table 3:** Some important physical and chemical properties of Heilu clay soil in Luochuan.

Soil layer code	Soil depth (m)	Bulk density (g/cm^3^)	Wilting moisture (m/m)	Field water-holding capacity (m/m)	Organic nitrogen (mg/kg)	pH	Organic matter (%)	Calcium carbonate (mg/kg)	Phosphorus (mg/kg)
1	0.01	1.18	0.10	0.31	350	8.1	0.75	4.4	3
2	0.5	1.23	0.10	0.29	380	8.1	0.90	1.5	3
3	1	1.33	0.10	0.30	360	8.1	0.91	1.4	3.2
4	2	1.35	0.10	0.30	370	8.2	0.85	1.03	3.2
5	3	1.34	0.11	0.32	380	8.2	0.83	11.3	3

**Table 4:** Some important modified parameters of apple growth used in WinEPIC.

Parameter	Definition	Value
CPNM	Crop name	APPLE
WA	Energy to biomass conversion factor (t/hm^2^.MJ^1^)	45.0
HI	Harvest index (crop yield/aboveground biomass usually valued at 0.01–0.95)	0.5
TG	Optimal temperature for plant growth (°C)	22.0
TB	Minimum temperature for plant growth (°C)	5.0
DMLA	Maximum potential leaf area index	3.5
DLAI	Fraction of growing season when the leaf area index starts to decline (usually valued at 0.4–0.99)	0.99
RLAD	Leaf area index decline rate parameter (usually valued at 0–10)	1.0
RBMD	Biomass-energy ratio decline rate parameter (usually valued at 0–10)	1.0
HMX	Maximum crop height (m)	4.0
RDMX	Maximum rooting depth (m)	10.0

### Simulation methods

In this study, 6 different fertilization treatments were applied to the local apple orchards on the same dates: F0 (N 0 kg/ha, P 0 kg/ha), F1 (N 120 kg/ha, P 60 kg/ha), F2 (N 240 kg/ha, P 120 kg/ha), F3 (N 360 kg/ha, P 180 kg/ha), F4 (N 480 kg/ha, P 240 kg/ha) and F5 (N 600 kg/ha, P 300 kg/ha). The soil water simulations commenced in the years when the apple trees were transplanted. In this simulation, the fertilizers were applied each year at a given time according to the fertilization treatment protocol. After the soil water parameters were entered in the model, the initial soil water condition was automatically set at 75% of the field water-holding capacity. Using WinEPIC, the daily dynamics of growth, soil water and fertilizer utilization from 1965 to 2009 were quantitatively simulated, and the daily biomass growth and soil water and nutrient balances were obtained. To fully reflect soil water consumption and the soil water balance conditions resulting from precipitation, the following was done: within the daily data sequence of simulated soil moisture in the 0–15 m layer, the data from the 15^th^ of each month were considered as characteristic of the available soil moisture for the month, and the soil moisture profile distribution data from the 1^st^ of November of each year were considered as characteristic for the year.

### WinEPIC model verification

**Figure 7:**
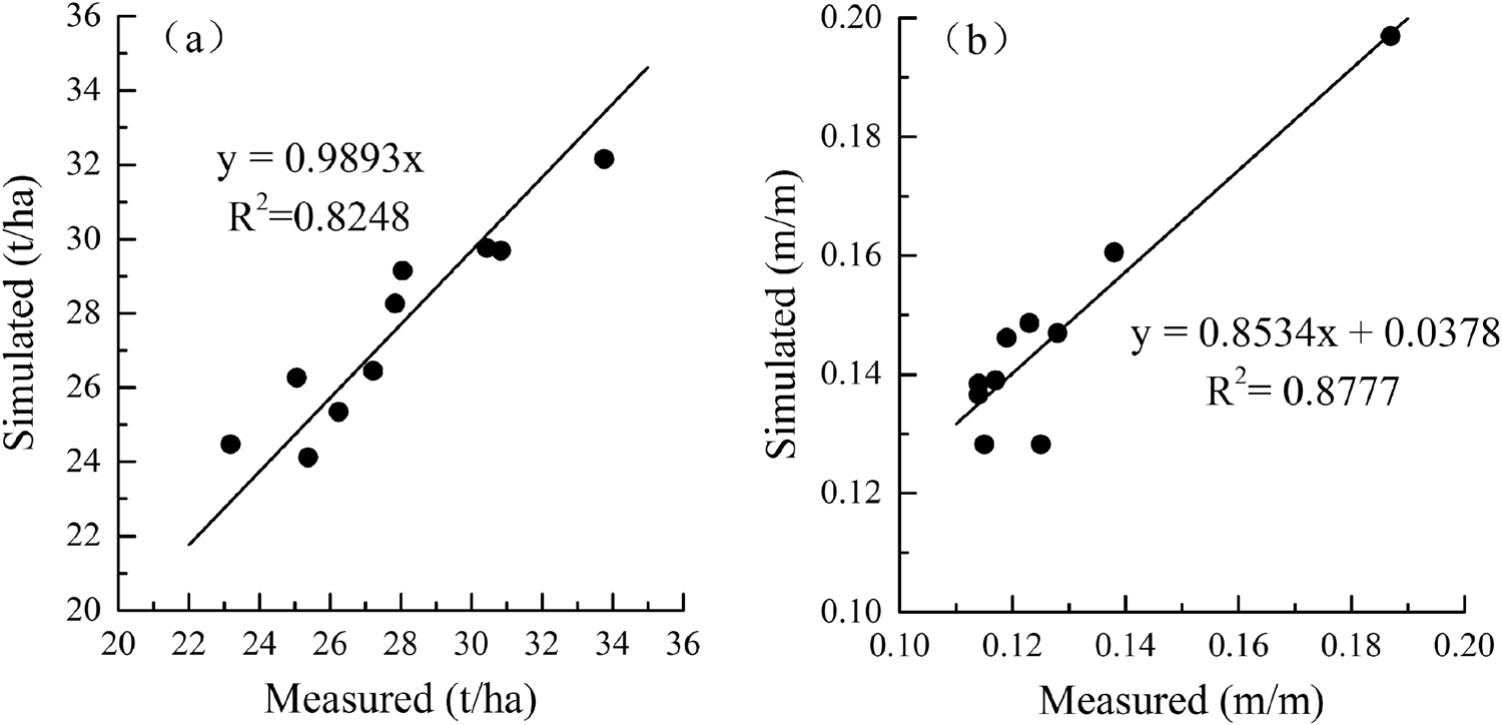
Simulated and measured yield (**a**) and soil moisture (**b**) in the rainfed apple orchards of Luochuan.

To verify the EPIC performance in simulating the yield and soil moisture of the dryland apple orchards, these values were simulated for apple orchards in Luochuan from 1980 to 2009. The differences between the simulated and measured yield and soil moisture (2–10 m) were compared to verify the performance of the EPIC model. The measured yields were adapted from orchard surveys conducted at different ages by the experimental station in Luochuan. A significant correlation was observed between the simulated and measured fruit yield in the apple orchards of Luochuan, with a relative deviation of 0.76%, an RMSE of 1.09 t/ha, and a correlation index of 0.94. With respect to soil water, the relative error was 13.1%, the RMSE was 0.019 m/m, and the correlation index was 0.97 (Table 5). In addition, the slope of the linear regression between the simulated and observed yield and soil water was close to 1, with an R^2^ value that showed a significant correlation (Fig. 7). EPIC revised with these parameters could be used to accurately simulate the yield response and soil moisture characteristics of dryland apple orchards in Weibei.

**Table 5:** Simulated and measured yield and soil moisture of apple orchards in Luochun.

	Fruit yield (t/hm^2^)		Soil moisture (m/m)	
	Measured	Simulated	Measured	Simulated
Average	27.57	27.80	0.147	0.128
RE (%)	0.76		13.096	
Correlation coefficient	0.940^**^		0.937^**^	
RMSE	1.090		0.019	
Regression equation	y = 0.9893x		y = 0.8534x + 0.0378	
R^2^	0.825		0.877

## References

[1] Wang J, Wu FQ, Meng QL (2006). Analysis on soil moisture character of dry orchard on hilly and gully regions on the Loess Plateau.J. Northwest For. Univ..

[2] Gong W, Yan XY, Wang JY (2011). Effect of long-term fertilization on soil fertility. Soils..

[3] Liu HJ, Ju XT, Tong YA, Zhang FS, Lv JL (2002). The status and problems of fertilization of main fruit trees in Shaanxi Province. Agric. Res. Arid Areas..

[4] Zhao ZP, Tong YA, Gao YM, Fu MM (2009). Effect of different fertilization on yield and quality of Fuji apple. Plant Nutr. Fert. Sci..

[5] Wang XY, Tong YA, Liu F, Zhao ZP (2013). Evaluation of the situation of fertilization in apple fields in Shaanxi province. Plant Nutr. Fert. Sci..

[6] Lu SC, Yan ZJ, Chen Q, Zhang FS (2012). Evaluation of conventional nitrogen and phosphorus fertilization and potential environmental risk in intensive orchards of North China. J. Plant Nutr..

[7] Wang R (2015). Phosphorus Accumulation and Sorption in Calcareous Soil under Long-Term Fertilization. Plos one.

[8] Wu FQ, Liu HB, Sun BS, Wang J, Gale WJ (2008). Net primary production and nutrient cycling in an apple orchard–annual crop system in the Loess Plateau, China: a comparison of Qinguan apple, Fuji apple, corn and millet production subsystems. Nutr. Cyc. Agroecosyst..

[9] Strapatsa AV, Nanos GD, Tsatsarelis CA (2006). Energy flow for integrated apple production in Greece. Agr. Ecosyst. Environ..

[10] Liu XZ, Wang Q, Yi HP (2006). Effects of Deep-ditch Fertilization Pattern on Soil Physical and Chemical Properties in an Apple Orchard. Chin. J. Soil Sci..

[11] Peng FT, Jiang YM (2006). Characteristics of N, P, and K nutrition in different yield level apple orchards. Sci. Agr. Sin..

[12] Dilini D, Syed KS, Hector M, Malka NH (2015). Root zone soil moisture prediction models based on system identification: Formulation of the theory and validation using field and AQUACROP data. Agric. Water Manage..

[13] Gong DZ, Kang SZ, Zhang L, Du TS, Yao LM (2006). A two-dimensional model of root water uptake for single apple trees and its verification with sap flow and soil water content measurements. Agric. Water Manage..

[14] Huang MB, Gallichand J (2006). Use of the SHAW model to assess soil water recovery after apple trees in the gully region of the Loess Plateau, China. Agric. Water Manage..

[15] Gaiser T, de Barros I, Sereke F, Lange FM (2010). Validation and reliability of EPIC model to simulate maize production in small-holder farming systems in tropical sub-humid West Africa and semi-arid Brazil. Agric. Ecosyst. Environ..

[16] Jones CA (1991). EPIC: an operational model for evaluation of agricultural sustainability. Agric.Syst..

[17] Williams JR, Jones CA, Kiniry JR, Spanel DA (1989). The EPIC crop growth model. Trans.ASAE.

[18] Ko J, Piccinni G, Steglich E (2009). Using EPIC model to manage irrigated cotton and maize. Agric. Water Manage..

[19] Michele R (2001). Application of EPIC model for irrigation scheduling of sunflower in Southern Italy. Agric. Water Manage..

[20] Wang XC, Li J, Tahir MN, Fang XY (2012). Validation of the EPIC model and its utilization to research the sustainable recovery of soil desiccation after alfalfa (Medicago sativa L.) by grain crop rotation system in the semi-humid region of the Loess Plateau. Agric. Ecosyst. Environ..

[21] Guo Z (2015). Simulation of water productivity and soil water use of different planting density apple orchard. Northern Horticul..

[22] Shan L (2002). Development Trend of dryland farming technologies. Sci. Agr. Sin..

[23] Holb I, Gonda I, Vago I, Nagy P (2009). Seasonal dynamics of nitrogen, phosphorus, and potassium contents of leaf and soil in environmental friendly apple orchards. Communications in soil science and plant analysis..

[24] Jun GB, Cha YL, Cao QH, Cheng SM (2009). The status and recommendations of fertilization of Fuji orchards in Changwu County. Shanxi Fruits..

[25] Cai Z (2011). Effects of long-term fertilization on pH of red soil, crop yields and uptakes of nitrogen, phosphorous and potassium. Plant Nutr. Fert. Sci..

[26] Fan J, Shao M, Hao M, Wang Q (2004). Desiccation and nitrate accumulation of apple orchard soil on the Weibei dryland. Chin. J. Appl. Ecol..

[27] Wang B (2007). Distribution features of soil water content in the profile of rainfed cropland with long-term fertilization. Plant Nutr. Fert. Sci..

[28] Tagliavini M, Marangoni B (2002). Major nutritional issues in deciduous fruit orchards of Northern Italy. HortTechno..

[29] Pacholak E (2008). Effect of 25 years of differentiated fertilization with NPK and magnesium on growth and fruit yield of apple ‘Cortland’ and on the content of minerals in soil and leaves. J. Fruit Ornam. Plant Res..

[30] Wang XC, Li J, Fan TL (2008). Modeling the effects of winter wheat and spring maize rotation under different fertilization treatments on yield and soil water in rain-fed highland of Loess Plateau. Plant Nutr. Fert. Sci..

[31] Wang XC, Li J, Hao MD (2008). Simulation of fertilization effect on winter wheat yield in Changwu dry highland. Trans. Chin. Soc. Agric. Eng..

[32] Williams JR (1990). The erosion productivity impact calculator (EPIC) model: a case history. Phil. Trans. Roy. Soc. Lond. B.

[33] Williams, J. R. The EPIC model. In: Singh, V. P. (Ed.), *Computer Models of Watershed Hydrology*. 909–1000 (Water Resources Publisher, Colorado, 1995).

[34] Li J, Shao MA, Zhang XC (2004). Simulation equations for soil water transfer and use in the EPIC model. Agric. Res. Arid Areas..

[35] Li J, Wang XC, Shao MA, Zhao YJ, Li XF (2008). Simulation of water productivity and soil desiccation effects of different planting density black locust forestlands on the Loess Plateau. Acta Ecol. Sin..

[36] Li J, Wang XC, Shao MA, Zhao YJ, Li XF (2010). Simulation of biomass and soil desiccation of Robinia pseudoacacia forestlands on semi-arid and semi-humid regions of China’s Loess Plateau. J. Plant Eco..

[37] Zhang SH, Li J, Wang XC, Wang YL (2011). Modeling the changes of yield and deep soil water in apple orchards in Weibei rainfed highland. Acta Ecol. Sin..

[38] Baier W, Robertson GW (1965). Estimation of latent evaporation from simple weather observation. Can. J. Plant Sci..

[39] Hargreaves GH, Samani ZA (1985). Reference crop evapotranspiration from temperature. Appl. Eng. Agric..

[40] Monteith, J. L. Evaporation and the environment. In: *The State and Movement of Water in Living organisms Xixth Symposium. Soc. for Exp.Biol*. **19**, 205–234 (Cambridge University Press, Swansea, 1965).

[41] Penman HL (1948). Natural evaporation from open water, bare soil, and grass. Proc. Roy. Soc. Lond. A.

[42] Priestley CHB, Taylor RJ (1972). On the assessment of surface heat flux and evaporation using large-scale parameters. Mon. Weather Rev..

[43] Li J, Shao MA, Zhang XC (2004). Database construction for the EPIC model on the Loess Plateau region. J. Northwest Sci-Tech Univ. Agr. For..

[44] The national soil survey and investigation office. *Records of Chinese soil survey*, Vol. 5, 244–254 (Chinese Agriculture Press, Beijing, 1995).

[45] Shaanxi province soil survey and investigation office. *Soils of Shaanxi province*. (Science Press, Beijing, 1992).

[46] Lu, Q. N. & Jia, D. X. *Chinese fruit trees, apple*. (Chinese forestry Press, Beijing, 1999).

